# Retrospective evaluation of pimobendan and sildenafil therapy for severe pulmonary hypertension due to lung disease and hypoxia in 28 dogs (2007–2013)

**DOI:** 10.1002/vms3.60

**Published:** 2017-03-08

**Authors:** Lisa A. Murphy, Nicholas Russell, Domenico Bianco, Reid K. Nakamura

**Affiliations:** ^1^ VCA Orange County Veterinary Specialists Tustin California USA; ^2^ Emergency and Cardiology Departments Veterinary Specialty and Emergency Center Thousand Oaks California USA; ^3^ Animal Medical Center of Southern California Los Angeles California USA

**Keywords:** Cardiology, Canine, Imaging‐Ultrasound

## Abstract

Pulmonary hypertension (PH) is the persistent abnormal increase in pulmonary artery (PA) pressure and in dogs is usually secondary to congenital disease causing pulmonary over circulation, chronic respiratory disease and elevated left atrial pressure. Sildenafil (SF) is a phosphodiesterase (PDE) V inhibitor that causes pulmonary artery (PA) vasodilation by increasing pulmonary vascular concentrations of cyclic guanosine monophosphate which subsequently increases the activity of endogenous nitric oxide. Pimobendan (PB) is a PDE III inhibitor with calcium sensitizing effects thereby exerting positive inotropy and vasodilation. The purpose of this retrospective study was to evaluate the long‐term survival of dogs with severe PH treated with SF and PB compared to SF alone. The use of PB in combination with SF did not result in a statistically significant increase in survival times in dogs with pulmonary hypertension secondary to chronic respiratory disease compared to SF alone.

## Introduction

Pulmonary hypertension (PH) is the persistent abnormal increase in pulmonary artery (PA) pressure (Johnson *et al*. 1999; Bach *et al*. 2006; Schober & Baade 2006; Brown *et al*. 2010). PH in dogs is usually secondary to congenital cardiac disease causing pulmonary over circulation and subsequent vascular remodelling, chronic respiratory disease or elevated left atrial (LA) pressure (Serress *et al*. 2006; Kellum & Stepien 2007; Kellihan & Stepien 2012). PH is not a specific disease but instead compromises a group of diseases which all cause elevated pulmonary artery pressure. The most recent consensus on PH in people occurred in 2013 and classified the syndrome into five classes (Fritz & Smith 2016). Class I, or pulmonary arterial hypertension, encompasses idiopathic disease. Class II, or pulmonary venous hypertension, is the most common form and is associated with left‐sided cardiac disease. Patients with PH from chronic pulmonary disease are categorized into class III while those with chronic thromboembolic disease are in class IV. Finally, diseases with poorly understood mechanisms precipitating PH are in class V.

Doppler echocardiography is considered the modality of choice for diagnosing PH in veterinary patients as it is readily available and non‐invasive (Johnson *et al*. 1999; Bach *et al*. 2006; Schober & Baade 2006; Serress *et al*. 2006; Brown *et al*. 2010). Peak tricuspid flow regurgitation velocity (PTFRV) can be ascertained via Doppler interrogation and be applied into the modified Bernoulli equation allowing systolic pulmonary artery pressure (sPAP) to be estimated (Johnson *et al*. 1999; Bach *et al*. 2006; Schober & Baade 2006; Serress *et al*. 2006; Brown *et al*. 2010). Dogs are classified as mild (sPAP < 50 mm Hg), moderate (sPAP = 50–75 mm Hg) and severe PH (sPAP > 75 mm Hg) (Johnson *et al*. 1999; Bach *et al*. 2006; Schober & Baade 2006; Serress *et al*. 2006; Brown *et al*. 2010). However, estimation of SPAP via the modified Bernoulli equation can be inaccurate with some papers reporting the estimated value to not correlate with hemodynamic measurements in 25% of patients while other studies report that this non‐invasive method underestimates PH in 47% of those evaluated and overestimates it in 13% (Denton *et al*. 1997; Fisher *et al*. 2009). Right heart catheterization (RHC) is considered the most accurate method for measurement of SPAP; however, this diagnostic modality is much more invasive and costly.

Sildenafil (SF) is a phosphodiesterase (PDE) V inhibitor that causes pulmonary artery (PA) vasodilation by increasing pulmonary vascular concentrations of cyclic guanosine monophosphate which subsequently increases the activity of endogenous nitric oxide (Bach *et al*. 2006; Kellum & Stepien 2007; Brown *et al*. 2010). Since 2005, SF has been licensed by the Food and Drug Administration for all functional classes of PH in adults (Chakrabarti *et al*. 2015). This was based on the results of SUPER‐1 trial which evaluated patients with PH from class II, III and IV and showed a reduction in the mean pulmonary artery pressure and the 6 min walk test compared to a placebo (Gaile *et al*. 2005). A post hoc analysis of the SUPER‐1 trial (Badesch *et al*. 2007) also supported SF use specifically in patients with parenchymal lung disease as it was shown to improve the results of the 6 min walking distance test after 12 weeks of treatment. However, there are also some concerns about the potential of SF to worsen arterial oxygenation via its affects on Ventilation/Perfusion mismatching when evaluating patients with class III PH (Blanco *et al*. 2010). These concerns stemmed from a trial showing SF inhibitinghypoxia‐induced pulmonary vasoconstriction in healthy subjects (Zhao *et al*. 2001). At this time, there is no consensus on the use of SF in the absence of severe PH in patients with class III PH (Seeger *et al*. 2013). Similarly, there is conflicting study results when trying to evaluate this medication in veterinary patients with PH and most of this information focuses on class II PH. Kellum & Stepien 2007 showed that there was no evidence of SF significantly affecting PTRFV, while another study (Bach *et al*. 2006) showed there was a small decrease in estimated SPAP. Similar to human medicine, however, it appears SF improves the clinical signs and quality of life in veterinary patients with PH (Kellum & Stepien 2007; Brown *et al*. 2010). Despite the initial clinical improvement, the long‐term outcomes in dogs with severe PH treated with SF in a small retrospective study is reportedly poor (Bach *et al*. 2006).

Pimobendan (PB) is a PDE III inhibitor with calcium sensitizing effects thereby exerting positive inotropic effects; it also causes systemic vasodilation (Böhm *et al*. 1991; Ohte *et al*. 1997). Experimental studies have demonstrated physiological benefits following PB administration in human patients with cor pulmonale (Nakatani *et al*. 1999) and chronic emphysema (Yamazaki *et al*. 1997) and there have been isolated reports of clinical improvement in humans with primary pulmonary hypertension that have been treated with PB in addition to other therapies (Watanabe *et al*. 2003; Sahara *et al*. 2006). However, inodilators are not considered first line therapy for human patients with class III PH (Seeger *et al*. 2013). Experimental PH in rats reported a reduced sensitivity to certain vasodilators, which may be due to increased PDE III and V and may explain the benefit of SF and PB in dogs with PH (Murray *et al*. 2002). In dogs with class II PH from myxomatous mitral valve disease, PB has been shown to lower the severity of measurable PTRFV, improve quality of life scores and decrease NT‐proBNP concentrations in the short term (Atkinson *et al*. 2009).The long‐term survival of dogs with severe class III PH treated with both PB and SF has not been previously reported. The purpose of this retrospective study was to evaluate the long‐term survival of dogs with severe class III PH from suspect chronic respiratory disease treated with SF and PB compared to SF alone.

## Materials and methods

Medical records were retrospectively reviewed for dogs diagnosed with severe PH between July 15th 2007 to March 1st 2013. The clinical protocol and data collection were approved by the institutional Veterinary Ethics and Review Committee. The echocardiogram had to be performed by a board‐certified cardiologist or a cardiology resident under supervision of a board‐certified cardiologist for inclusion into the study. Dogs had to have a PTRFV of >4.33 m/sec for inclusion into the study. Dogs were excluded if their type of PH was classes I, II, IV and V and thus not suspected to be from chronic respiratory disease (class III). Medical records were reviewed for the following information: signalment, clinical signs, physical examination findings and results of diagnostic testing. The dosage and dosing of SF (Viagra, Pfizer U.S. Pharmaceuticals, New York, NY) and PB (Vetmedin, Boehringer Ingelheim Pharma KG, Ingelheim, Germany), effect of treatment on clinical signs and PTRFV, and survival time from diagnosis was also recorded. Survival data were calculated from the day of diagnosis of severe PH. When follow‐up data was not available, a phone conversation to the regular veterinarian or owner was made. Dogs with pulmonic stenosis, right ventricular (RV) outflow tract obstruction or without severe PH were excluded from analysis. Dogs not treated with SF were also excluded from analysis as well.

### Statistical analysis

Data from the dogs in the two groups were compared by use of a Student t‐test for data with normal distribution and a Mann–Whitney rank sum test was used for data with non‐normal distribution. The Kaplan–Meier estimates of the distribution of times from diagnosis to death were computed, and the Mantel‐Cox log‐rank analysis was performed to compare the survival curves between the two groups. Results are presented as median and range unless indicated otherwise. Statistical analyses were performed using a standard statistical software package (SPSS 22.0 for Windows, Microsoft, Redmond, WA). For all analyses, values *P* < .05 were considered statistically significant.

## Results

Forty dogs were identified with severe PH during the study period. Five dogs were excluded because they were not treated with SF therapy. Three dogs were diagnosed with severe class I PAH secondary to pulmonary over‐circulation (one dog was diagnosed with a perimembranous ventricular septal defect and two dogs were diagnosed with a patent ductus arteriosus) and were excluded as well. Four additional dogs were excluded as the PH was associated with elevated LA pressure from severe myxomatous mitral valve disease. There were a total of 28 dogs identified with severe class III PH secondary to chronic respiratory disease. There were 16 dogs treated with PB and SF simultaneously and designated the treatment group and there were 12 dogs treated with SF only and designated the control group.

There was no significant difference between the two groups with regard to age and weight (Table 1). The breeds identified in the treatment group included Chihuahua (*n* = 3), West Highland white terrier (*n* = 3), Pomeranian (*n* = 2), Terrier (*n* = 2), Shih Tzu (*n* = 2) and one dog each of the following: Cavalier King Charles Spaniel, Pit Bull, Miniature Schnauzer, and Toy Poodle. Breeds identified in the control group were Chihuahua (*n* = 4), Pomeranian (*n* = 2), Mix breed (*n* = 2) and one dog each of the following: Labrador retriever, Shih Tzu, Australian Shepherd and Yorkshire terrier. There were seven spayed females, six male castrated, two male intact and one female intact in the treatment group and there were 10 spayed females, one male castrated and one female intact dog in the control group. There were no significant differences between the two groups with regards to presenting complaints or physical examination findings (Tables 1, 2, and 3).

**Table 1:** Signalment and clinical signs of treatment and control groups

Category	Treatment group (*n* = 16)	Control group (*n* = 12)	*P* value
Age (months)	136 (93–216)	140 (96–176)	0.82
Weight (kgs)	7.70 (1.44–27.82)	4.75 (1.63–29.09)	0.39
Coughing	8/16	7/12	0.78
Abdominal Distension	5/16	0/12	0.18
Respiratory Distress	13/16	10/12	0.73
Lethargy	8/16	5/12	0.67
Syncope	2/16	4/12	0.64
Exercise Intolerance	8/16	6/12	1.0

**Table 2:** Physical examination findings of treatment and control groups

Category	Treatment group (*N* = 16)	Control group (*N* = 12)	*P* value
Heart rate (bpm)	130 (80–164)	130 (100–140)	0.89
Respiratory description	Dyspnoeic = 8/14	Dyspnoeic = 5/11	0.41
	Tachypneic = 3/14	Tachypneic = 2/11	0.82
	Panting = 1/14	Panting = 4/11	0.29
	Eupneic = 2/14	Eupneic = 0/11	0.78
	Not described = 2	Not described = 1	N/A
Crackles	2/16	6/12	0.23
Harsh or increased respiratory sounds	14/16	11/12	0.92
Cyanosis	3/16	7/12	0.44

**Table 3:** Treatment and outcome

	Treatment Group (*n* = 16)	Control Group (*n* = 12)	*P* value
Initial sPAP (mm Hg)	104.19 mm Hg (78–194.79 mm Hg)	90.4 mm Hg (75.3–159.29 mm Hg)	0.86
Sildenafil dose (mg/kg)	1.54 mg/kg (1.00–2.98 mg/kg)	1.84 (0.66–2.93 mg/kg)	0.91
Other therapy	Antibiotics = 7/16	Antibiotics = 7/12	1.00
	Airway dilators = 3/16	Airway dilators = 4/12	0.75
	Diuretics = 3/16	Diuretics = 1/12	0.33
Clinical status	Improved, still tachypneic = 5/9	Improved, still tachypneic = 6/9	0.92
	Improved seems normal = 3/9	Improved seems normal = 2/9	0.88
	Worsened = 1/9	Worsened = 1/9	1.00
Reduction in sPAP	22.02 mm Hg (range 1.5–103.66 mm Hg)	24.70 mm Hg (18–53 mm Hg)	0.89
Survival time	102 days Range (1–390 days)	44.5 days (1–378 days)	0.51

Complete blood counts were performed in eight dogs in the treatment group and were normal in seven dogs and the last showed a neutrophilia. Complete blood counts were performed in seven dogs in the control group and were normal in five dogs and showed a neutrophilia in the remaining two. Serum chemistry profile was performed in 10 dogs in the treatment group and showed liver enzyme elevations in two dogs, azotemia in two dogs and was normal in the remaining dogs. Serum chemistry profile was performed in eight dogs in the control group and revealed elevated liver enzymes in three dogs, azotemia in one dog and hyperglycemia in one dog. D‐dimer testing was performed in three dogs in the treatment group and two dogs in the control group and was negative in all dogs. Heartworm testing was performed in three dogs in the treatment group and two dogs in the control group and was negative in all dogs. Three dogs in the treatment group and one dog in the control group had their systemic blood pressure measured indirectly by Doppler method and was normal in all dogs. Electrocardiography was performed in was performed in three dogs in the treatment group and a respiratory sinus arrhythmia was identified in two dogs and normal sinus rhythm was identified in the remaining dog. Electrocardiography was not performed in any dogs in the control group. Radiographic abnormalities in the treatment group included diffuse interstitial pulmonary parenchyma (*n* = 10), right sided cardiomegaly (*n* = 8), generalized cardiomegaly (*n* = 3), enlarged PA (*n* = 3) and focal infiltration in the left lung field (*n* = 1). Radiographic abnormalities in the control group included right‐sided cardiomegaly (*n* = 5), diffuse interstitial pulmonary parenchyma (*n* = 5), generalized cardiomegaly (*n* = 3), focal infiltration in the right lung field (*n* = 1), enlarged PA (*n* = 1) and a pleural fissure line (*n* = 1).

Complete echocardiographic examinations were performed in all 28 dogs. The left atrium to aorta ratio (LA/Ao) based on two‐dimensional measurement of the treatment group (1.33, range 0.9–1.6) was not statistically different compared to the control group (1.16, range 0.95–1.54) (*P* = 0.91). In the treatment group, the left ventricle (LV) was assessed as small in 11 dogs, normal in size in four dogs, and mildly dilated in one dog. In the control group, the LV was assessed as small in two dogs, normal in eight dogs, mildly dilated in one dog and moderately dilated in one dog. In the treatment group, the right atrium (RA) was subjectively assessed as severely enlarged in eight dogs, moderately enlarged in seven dogs and normal in one dog. In the control group, the RA was subjectively assessed as severely enlarged in seven dogs, moderately enlarged in three dogs and mildly enlarged in two dogs. The RV was assessed as severely enlarged in eight dogs, moderately in seven dogs and normal in one dog in the treatment group. In the control group, the RV was assessed as severely enlarged in 5 days, moderately enlarged in five dogs, mildly enlarged in 1 dog and normal in 1 dog. Septal flattening was identified in 15 dogs in the treatment group and in nine dogs in the control group. Paradoxical septal motion was identified in eight dogs in the treatment group and in two dogs in the control group. The PA was dilated in seven dogs in the treatment group with seven dogs showing evidence of pulmonic insufficiency, classified as mild in six dogs and moderate in one dog. In the control group, the PA was dilated in four dogs with three dogs having mild pulmonic insufficiency. The PA flow profile in the treatment group was assessed as type II in eight dogs and type III in the remaining eight dogs. The PA flow profile in the control group was type II in six dogs and type III in six dogs. Pleural effusion was identified in two dogs in the treatment group. In the control group, one dog has mild pleural effusion identified and another dog had mild pleural and pericardial effusion identified.

The median PTRFV was not statistically different between the treatment (5.09 m/s, range 4.41–6.97 m/s) and control group (4.75 m/s, range 4.33–6.31 m/s) (*P* = 0.86). There was no statistically significant difference between the median sildenafil dose between the treatment (4.62 mg/kg/d divided three times, daily range 3.00–5.96 mg/kg/d) and control group (5.52 mg/kg/d divided three times, daily range 1.98–8.79 mg/kg/d) (*P* = 0.91). The median pimobendan dose utilized in the treatment group was 0.28 mg/kg (range 0.20–0.46 mg/kg twice a day). Other therapy used for dogs in the treatment group included antibiotics (*n* = 7), airway dilators (*n* = 3), and diuretics (*n* = 3). For the control group, other treatment included antibiotics (*n* = 7), airway dilators (*n* = 4), diuretics (*n* = 1) and aspirin therapy (*n* = 1). Five dogs in each group were hospitalized, in both groups the median hospitalization time was 2 days, and in both groups 3 of the five dogs survived to discharge.

Nine dogs in each group had a recheck examination performed. In the treatment group, the median time to recheck examination was 14 days (range 7–60 days). Five dogs were described as improved but still tachypneic, three dogs were improved to the point of appearing normal to the owner and one dog appeared clinically worse to the owner. In the control group, the median time to recheck examination was 14 days (range 7–90 days). Six dogs were described as improved but still tachypneic, two appeared improved to the point of appearing normal and one dog appeared clinically worse to the owner. The median reduction in sPAP was not statistically different between the treatment (22.02 mm Hg, range 1.5–103.66 mm Hg) and control group (24.70 mm Hg, range 18–53 mm Hg) (*P* = 0.89). The median survival time of the treatment group was not statistically different (102 days, range 1–390 days) compared to the control group (44.5 days, range 1–378 days) (*P* = 0.51).

## Discussion

The primary finding of this study was that dogs with severe class III PH treated with PB in addition to SF did not have different survival times to dogs treated with SF alone. The median survival time of dogs treated with PB and SF in this study was also comparable to the median survival time reported previously for dogs treated with SF alone, however, this study focused on only dogs with severe class III PH (Bach *et al*. 2006). Although it may be difficult to extrapolate from studies in human patients, the most recent European Society of Cardiology and the European Respiratory Society guidelines stated that there was no specific therapy proven for the treatment of class III PH; however, long‐term oxygen administration may help slow down the progression of the disease. These guidelines also questioned the use of accepted therapies for class I disease, like SF, for people with pulmonary venous hypertension from left‐sided heart failure. (Galie *et al*. 2015). Unfortunately, for veterinary patients, long term at home oxygen administration is not available for most owners and while there are newer human medications being evaluated for this syndrome, these have not been tested in canine patients as their use is cost prohibitive at this time.

PTRFV was used in this case to diagnose severe PH although there are other means of diagnosing PH echocardiographically including systolic time intervals, main PA:Ao ratio, and end diastolic pulmonary insufficiency jet velocity (Kellihan & Stepien 2012). Each criteria has varying sensitivities and specificities for identifying PH, but they are unable to correlate abnormalities to severity of PH. As such they were not used for the inclusion criteria in this study. As discussed earlier, RHC is considered the gold standard in human medicine however is typically invasive and expensive (Martin‐Duran *et al*. 1986; McLaughlin *et al*. 2009). As such it is not routinely performed in the diagnosis of PH in dogs and was not performed for any dog included in this study. The authors utilized the PTRFV to help identify animals with suspect class III PH.

The majority of animals in this study were treated with concurrent medications, including diuretics, antibiotics and bronchodilators. The effect of these drugs on the treatment of PAH and outcome in each individual case is unclear. Theophylline is a weak, non‐selective PDE inhibitor with effects on PDE III, PDE IV and PDE V. However, its inhibitory effects on these receptors are dose dependent and the percentage of inhibition is small in humans with therapeutic drug concentrations (Poison *et al*. 1978). Furosemide was the only diuretic utilized in this cohort of patients because of the development of right sided heart failure (i.e. pleural effusion and ascites) and is not associated with beneficial effects for management of PH secondary to chronic respiratory diseases as the patients in this series exhibited. Broad spectrum antibiotics were used empirically in several of these patients in an attempt to ameliorate their severe clinical signs, but acute pulmonary infectious disease was not suspected in any of these patients. Diagnostic testing with bronchoalveolar lavage or transtracheal wash would have been ideal however most of the animals in this study were dyspnoeic and in a critical condition and would not have been safe anaesthetic candidates. One dog that had shown good response to treatment for PH with SF and PB underwent bronchoscopy and transtracheal wash for complete respiratory evaluation and died shortly after the procedure. Furthermore, bronchoalveolar lavage has been reported to inconsistently identify the underlying respiratory pathology (Hawkins *et al*. 1995; Hawkins *et al*. 1993) and thus is no longer routinely performed in dogs with severe PH at the authors’ institution.

Necropsies were not performed in any of the animals that died during the study period so authors were unable to confirm death due to PAH. However, most dogs died or were killed because of continued respiratory distress and thus it seems most likely the symptoms were secondary to the severe PAH identified on echocardiogram. No other comorbidities were identified which would explain the dogs’ demise.

Other limitations of this study include lack of a control group, lack of standardized dosing regimens for SF and PB, and inconsistent intervals between medication administration and PTFRV measurement for dogs with a recheck examination performed. Most dogs in this study did not have D‐dimer or heartworm antigen testing. Heartworm is very rare in the area where this study was performed, but it cannot be completely ruled out as an underlying cause for the severe PH in some of the dogs reported here. Blood work was performed in eight dogs, included a Complete Blood Count and Biochemistry and no significant findings were noted in the majority of these dogs, so an underlying disease predisposing dogs to thromboembolic disease appears less likely but cannot be completely ruled out. Although D‐dimers were assessed in three dogs, their use for diagnosing PTE are not without limits as values may peak at 2 h post thromboembolic before declining in the subsequent 24 h (Goggs *et al*. 2014). Even if elevated D‐dimer values are identified in an animal, more recent studies (Epstein *et al*. 2013; Goggs *et al*. 2014) have shown a poor correlation between increased D‐dimer concentrations and PTE. A recent study found that thoracic computed tomography appears more sensitive than D‐dimer testing for the detection of PTE (Goggs *et al*. 2014), however, was not available at the authors’ institution at the time of this study. The diagnostic value of D‐dimers for dogs with severe PH remains unclear at this time.

## Conclusion

In conclusion, dogs with severe class III PH found no benefit when treated PB in addition to SF therapy. There were no discernible adverse effects associated with the administration of SF and PB in dogs with severe PHT. Prospective randomized controlled studies are warranted to evaluate these initial results.

## Source of funding

There was no external sources of funding for this study.

## Conflict of interest

The authors declare no conflicts of interest.

## Contributions

There are no contributions to declare.
